# Predicting Diffusion Coefficients in Nafion Membranes during the Soaking Process Using a Machine Learning Approach

**DOI:** 10.3390/polym16091204

**Published:** 2024-04-25

**Authors:** Ivan Malashin, Daniil Daibagya, Vadim Tynchenko, Andrei Gantimurov, Vladimir Nelyub, Aleksei Borodulin

**Affiliations:** 1 Artificial Intelligence Technology Scientific and Education Center, Bauman Moscow State Technical University, 105005 Moscow, Russiaagantimurov@emtc.ru (A.G.); vladimir.nelub@emtc.ru (V.N.); alexey.borodulin@emtc.ru (A.B.); 2 P.N. Lebedev Physical Institute of the Russian Academy of Sciences, 119991 Moscow, Russia; 3 Scientific Department, Far Eastern Federal University, 690922 Vladivostok, Russia

**Keywords:** Nafion, IR spectroscopy, machine learning, diffusion, neural networks, fine-tuning optimization, genetic algorithm

## Abstract

Nafion, a versatile polymer used in electrochemistry and membrane technologies, exhibits complex behaviors in saline environments. This study explores Nafion membrane’s IR spectra during soaking and subsequent drying processes in salt solutions at various concentrations. Utilizing the principles of Fick’s second law, diffusion coefficients for these processes are derived via exponential approximation. By harnessing machine learning (ML) techniques, including the optimization of neural network hyperparameters via a genetic algorithm (GA) and leveraging various regressors, we effectively pinpointed the optimal model for predicting diffusion coefficients. Notably, for the prediction of soaking coefficients, our model is composed of layers with 64, 64, 32, and 16 neurons, employing ReLU, ELU, sigmoid, and ELU activation functions, respectively. Conversely, for drying coefficients, our model features two hidden layers with 16 and 12 neurons, utilizing sigmoid and ELU activation functions, respectively.

## 1. Introduction

Nafion [1], a widely used polymer in various applications such as electrochemistry [2], fuel cells [3], and membrane technologies [4], exhibits complex behaviors in saline environments, particularly regarding swelling and drying processes. Gaining insights into these phenomena holds paramount importance for optimizing the performance of Nafion across diverse implementations.

### 1.1. Nafion in Salt Solutions

**Figure 1:**
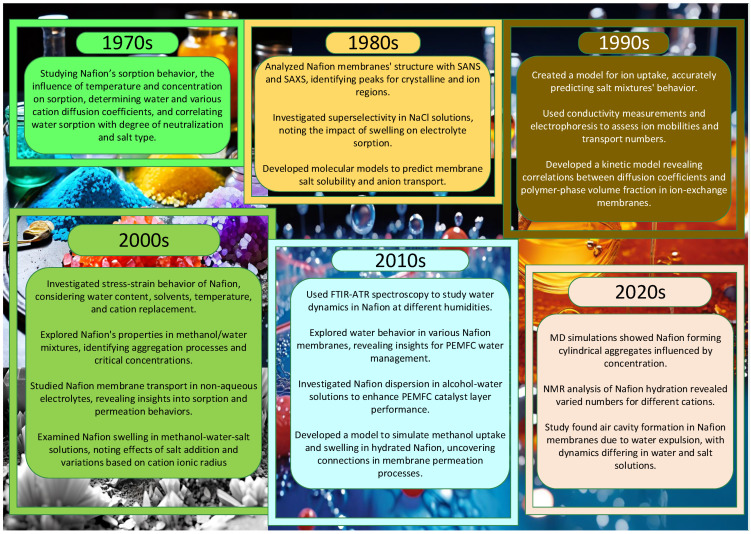
Retrospective overview of Nafion research in salt solutions: highlights of key topics from top scientific articles from each decade.

The investigation of Nafion in salt solutions is extensively covered in many literary sources over the last six decades, encapsulating key insights highlighted in the diagram depicted in Figure 1. One of the first [5] articles in the realm of this field of science investigates the sorption behavior of water and aqueous salt solutions of Nafion and reveals temperature and concentration dependencies of sorption, apparent activation energies for water diffusion in different membrane forms, and a maximum in sorption curves during neutralization, attributed to differences in diffusion coefficients. Additionally, diffusion coefficients of various cations were determined, showing a linear relationship with the charge-to-separation ratio, and water sorption was found to be strongly dependent on both the degree of neutralization and salt type, with a linear correlation between the number of water molecules absorbed and the degree of neutralization for all salts studied.

Measurements of densities and expansion coefficients of various Nafions in both acid and salt forms were provided in [6], investigating the effects of equivalent weights, moisture contents, and uniaxial orientations. Densities appeared to be independent of the equivalent weight but highly dependent on moisture content, with thin films showing strong uniaxial orientation. Reproducible expansion coefficients were observed after annealing, with distinct breaks in the linear expansion curve correlating with the material’s glass transition and mechanical dispersion. The experimental scatter in densities suggests possible microphase separation or partial crystallization of the polymer.

The physical structure of Nafion membranes in [7] was probed using small-angle neutron scattering (SANS) and small-angle X-ray scattering (SAXS). While acid-form samples exhibit two scattering peaks corresponding to crystalline and ion-containing regions, amorphous salt-form samples lack the first peak, allowing for a detailed investigation of the second peak, indicative of water-swollen regions.

Experimental measurements, including zero-current membrane potential, electrolyte sorption, self-diffusion fluxes of co-ions and counter-ions, co-ion fluxes under constant current, and membrane electrical conductance, were conducted in [8] to investigate the factors contributing to the superselectivity of Nafion membranes in NaCl solutions. Swelling was found to influence these factors, with expanded membranes exhibiting higher electrolyte sorption. However, the molarity of the sorbed electrolyte remained unchanged as swelling increased. The loss of superselectivity was attributed to a decrease in the mobility ratio of counter ions to co-ions.

The “cluster network” structure of Nafion membranes was utilized in [9] to develop models for membrane salt solubility and anion transport. Salt uptake was influenced by the size and charge of membrane clusters, while anion transport parameters depended on the membrane tortuosity and electrostatic forces in the interconnected pores. The models accurately predicted cation concentrations and the concentration dependence of anion–membrane interaction parameters.

A molecular-level equilibrium partition coefficient model has been developed in [10] to describe the uptake of single and multicomponent ions by a Nafion 117 cation exchange membrane. The model assumes a cylindrical pore structure and considers changes in ion solvation free energy during partitioning, as well as the orientation of solvent dipoles within the membrane pores due to the strong electric field from fixed charge groups. Membrane structure parameters, derived from experimental data on membrane porosity and X-ray diffraction, are used to predict equilibrium cation concentrations within a 6% accuracy for various aqueous salt mixtures. Analysis of computed electrostatic and hydration forces suggests that ions with a higher surface charge density are excluded from the pore wall region, similar to co-ions.

The overall transport characteristics of various cationic species in Nafion^®^ 117 membranes, including equilibrium salt and solvent uptake to determine membrane chloride concentration, porosity, and water content, were presented in [11]. Conductivity measurements via impedance spectroscopy were conducted for sodium and proton cationic forms, while the electrical mobilities of sodium, nickel, and silver ions were determined using electrophoresis. Additionally, combining these results allowed for the determination of the sodium transport number in the Nafion^®^ 117 membrane equilibrated with NaCl solution.

A sophisticated kinetic model is developed in [12] to study the coupled diffusion of two counterions in an ion-exchange membrane, considering the impact of varying the ionic composition on the membrane water content. Through numerical simulations, diffusion coefficients of alkali metal cations in different Nafion forms are evaluated, revealing a correlation between the membrane-to-aqueous ion diffusion coefficient ratio and the polymer-phase volume fraction. This study suggests that while the polymer phase mainly exhibits a steric effect, differences in the behavior between Nafion forms may be attributed to distinct morphologies.

Analysis of Nafion membranes through a dynamic mechanical analysis and tension film measurements was described in [13], observing a decrease in the initial slope of stress–strain curves with an increasing water content and the addition of certain solvents, and a decrease with increasing temperature. Additionally, the initial slope was found to increase with the replacement of cations, following the order Li^+^, Na^+^, K^+^, Cs^+^, and Rb^+^. Nafion in salt form typically exhibits an initial slope increase up to around 90 °C, followed by a decrease with rising temperatures.

The dilute solution properties of Nafion in methanol/water mixtures was studied in [14], revealing two aggregation processes: primary aggregation forming smaller, rod-like particles (<103 nm) due to hydrophobic interactions, and secondary aggregation forming larger particles (104 nm) attributed to ionic interactions. Critical concentrations of Cp (1.0 mg/mL) and Cpp (5.0 mg/mL) mark transitions in Nafion aggregation conformations, representing the formation of secondary ionic aggregations and the onset of self-assembly of disordered segments, respectively.

Paper [15] highlighted that the study of transport phenomena across ion-exchange membranes in non-aqueous electrolyte solutions remains limited, despite extensive research in aqueous systems that significantly increased the understanding of transport in aqueous–organic electrolyte solutions, particularly for applications like direct methanol fuel cells. Investigating Nafion membrane behavior in methanol–water electrolytes aims to shed light on its sorption and permeation properties, especially in the context of different electrolyte compositions.

Using the optical method, paper [16] examined how Nafion 112 foil swells anisotropically in methanol–water–inorganic salt solutions. Their findings indicate that even small amounts of inorganic salt in a methanol–water mixture affect both the rate and extent of swelling. We studied the effects of various inorganic salts, including LiCl, NaCl, KCl, CsCl, CaCl_2_, CdCl_2_, $K_{2}$${\mathrm{CO}}_{3}$, ${\mathrm{KNO}}_{3}$, ${\mathrm{NH}}_{4}$Cl, and ${\mathrm{AgNO}}_{3}$. The swelling kinetics of Nafion in ternary mixtures containing salt exhibited a maximum, suggesting that initially, methanol diffusion is faster than ion transport. This experimental data suggest that the swelling of Nafion decreases with an increase in the ionic radius of the cation.

Employing time-resolved Fourier transform infrared–attenuated total reflectance (FTIR-ATR) spectroscopy to investigate water dynamics in Nafion at low and high humidities was evaluated in [17]. At low humidities, non-Fickian behavior is observed due to a reaction between water and sulfonic acid, while at high humidities, water-induced relaxation in the polymer backbone leads to non-Fickian behavior. A diffusion–reaction model and a diffusion–relaxation model were developed, providing valuable insights into water transport mechanisms and relaxation phenomena in solid-state polymer electrolytes like Nafion.

Water absorption, swelling, and self-diffusivity in 1100 equivalent weight Nafion were analyzed across different temperatures and water activities in [18]. The study revealed a decrease in free volume per water molecule and a transition in the diffusivity rate at a water activity of four. Changes in hydrophilic domain connectivity were observed, informing the determination of interfacial mass transport coefficients and the development of a diffusion model for resolving water activity profiles.

An investigation of the swelling and diffusion behavior of a Nafion^®^ 117 ion-exchange membrane in mixed water–methanol solutions was presented in [19], analyzing the membrane porosity and water/methanol uptake using Raman spectrometry. Permeation experiments, considering adjacent diffusion boundary layers (DBLs) and the methanol diffusion coefficient’s dependency on concentration, were conducted under various conditions. Numerical fitting of experimental data determined the DBL thickness and methanol permeability, revealing the significant impact of DBL diffusion resistance on overall permeability, even at high rotation speeds. This study derived an equation relating the apparent non-electrolyte permeability to the true membrane permeability and diffusion layer thickness, following Helfferich’s approach.

The optimization of water management in proton exchange membrane fuel cells is crucial for advancing this technology. One study [20] utilized SANS to investigate water sorption processes, revealing flat water concentration profiles across the membrane under different equilibration conditions. The rapid swelling kinetics of a Nafion membrane immersed in liquid water, completing in less than a minute, is also reported for the first time, offering valuable insights into membrane behavior.

Exploration of water uptake and salt transport across different Nafion membranes in various aqueous salt solutions was investigated in [21], showing that water uptake increases with membrane thickness and decreases with cation size. The results also reveal a decrease in the integral permeability coefficient with membrane thickness, while the effect of electrolyte type on this coefficient is minimal for thicker membranes.

The impact of water/alcohol composition on the dispersion of H-Nafion^®^ in water/1-propanol and water/ethanol solutions was described in [22], which is crucial for catalyst layer performance in proton exchange membrane fuel cells (PEMFCs). Utilizing dynamic light scattering (DLS), small-angle X-ray scattering (SAXS), and nuclear magnetic resonance (NMR) spectroscopy, this paper reveals that 1-propanol induces notable changes in rod-like particle characteristics, suggesting implications for enhancing PEMFC performance.

Diffusion within polymer electrolyte membranes is often concurrent with time-dependent processes like swelling and polymer relaxation, limiting their ability to prevent molecular crossover during operation. To explore the coupling of such changes with the permeation process, a stochastic multiscale reaction–diffusion model was developed in [23], simulating methanol uptake and the swelling of hydrated Nafion. Comparison with experimental data suggests that a reaction-limited local response to an increasing methanol concentration best matches the observed behavior, indicating that interactions between methanol and Nafion lead to increased permeation across the membrane.

Coarse-grained molecular dynamics simulations elucidated the self-assembly behavior of Nafion ionomers in 1-propanol (NPA)/water solutions, revealing the formation of cylindrical aggregates with diameters of 2–3 nm [24]. The size and morphology of these aggregates were observed to vary nonlinearly with the ionomer concentration and NPA/water fraction, influenced by electrostatic repulsion among sulfonate groups and modified significantly upon salt addition, forming larger disk-shaped and secondary aggregates.

The hydration behavior of Nafion 117 membranes in alkaline ion forms was examined using high-resolution ^1^H NMR [25], revealing different hydration numbers for Li^+^, Na^+^, and Cs^+^ cations [26]. Cation self-diffusion coefficients, measured for the first time using pulsed-field gradient NMR, displayed a trend of Li^+^ ≤ Na^+^ > Cs^+^, with distinct activation energies indicating differences in diffusion behavior. Ionic conductivities calculated from these coefficients closely matched experimental values obtained via impedance spectroscopy, but with slightly higher estimations.

The study in [27] examines the behavior of Nafion membranes when swelling is limited to spaces smaller than 300 µm, leading to the formation of air cavities due to the expulsion of water from swollen polymer fibers. Investigating the collapse dynamics of these cavities in deionized water [28] and aqueous salt solutions reveals differing characteristic times, suggesting potential implications for pharmaceutical preparation processes requiring standardized dilution protocols.

### 1.2. Nafion and ML

**Figure 2:**
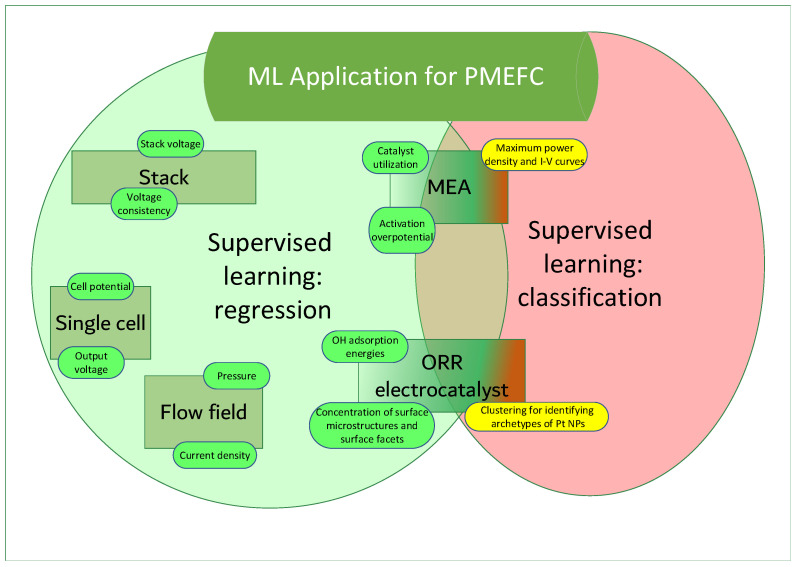
ML methods in PEMFCs.

ML presents a promising approach to optimize PEMFC performance, addressing challenges in cost and efficiency. By extracting patterns from experimental or simulation data, ML can predict outputs and reduce both experimental and computational costs. ML has demonstrated success in tasks such as predicting active electrocatalysts; optimizing membrane electrode assembly (MEA) [29], especially for predicting catalyst utilization [30], activation overpotential [31], maximum power densities, and I-V curves [32]; as an oxygen reduction reaction (ORR) electrocatalyst [33], especially regarding OH adsorption energies [34,35], concentration of surface microstructures and surface facets and clustering for identifying archetypes of Pt NPs [33]; and designing efficient flow channels [36], revolutionizing research in this field. Paper [37] reviews ML applications in optimizing PEMFC performance, offering insights for newcomers and outlining future directions for development. Figure 2 showcases the applications of ML methods in PEMFCs for enhancing performance and efficiency.

Study [38] utilized ML to analyze a database comprising 789 data points from 30 recent publications on polymer electrolyte membrane (PEM) electrolysis. Box whisker plots identified factors such as pure Pt at the cathode surface, Ti at the anode support, and specific compositions at the anode surface that led to higher performance. Principal component analysis (PCA) [39] highlighted risk factors for a low performance, including certain compositions at the cathode and anode surfaces. Classification trees identified the cathode-surface Ni mole fraction and the anode-surface Co mole fraction as crucial variables for electrolyzer performance, while regression trees successfully modeled the polarization behavior with an RMSE value of 0.18.

ML models, including polynomial and logistic regression, were proposed in [40] to predict the optimal design of PEM electrolyzer cells. Trained on 148 samples and validated on 16, the models accurately predicted eleven parameters based on inputs such as the hydrogen production rate and cell design. Hydrogen production rates measured using a custom-made PEM electrolyzer cell closely matched the simulation results, indicating the effectiveness of the models. This approach offers a cost-effective and time-saving solution for developing water electrolyzer cells for future hydrogen production.

Modern industries are increasingly adopting hydrogen as an energy carrier to decarbonize the electricity grid. However, the advancement of PEMFCs, which use hydrogen to produce electricity, is hindered by unpredictable performances and failure events like flooding and dehydration. To address this, paper [41] proposes a machine learning model to predict these failure modes by analyzing cell voltage and current density data. Utilizing advanced regression techniques like support vector machine, Decision Tree Regression, Random Forest Regression, and artificial neural networks, this model accurately forecasts flooding- and dehydration-induced failure events based on features derived from cell polarization data. Validation with real-time test data confirms the model’s reliability.

### 1.3. Aim of this Study

A significant body of research has been dedicated to investigating Nafion in salt solutions, spanning over several decades and encompassing various aspects such as sorption behavior, structural characterization, and ion transport properties. Despite the extensive use of machine learning (ML) in various aspects of Nafion-based PEMFCs, its application to predict diffusion coefficients for Nafion membranes based on infrared spectroscopy (IR) data remains unexplored. This presents a significant gap in our understanding, as accurate prediction of diffusion behavior is crucial for optimizing the performance and durability of PEMFCs. Therefore, the objective of this research is to fill this gap by leveraging the IR spectra of Nafion membranes to develop ML models capable of predicting diffusion coefficients accurately. By harnessing the information contained within IR spectra, we aim to advance our understanding of Nafion membrane ion-exchange behavior.

## 2. Materials and Methods

In our study, we employed Nation N117 cast films (Sigma Aldrich, St. Louis, MI, USA) with a thickness of 175 μm and an area of 1 × 1 cm^2^. The experimental setup utilized an analytical Fourier spectrometer FSM 2201 (LLC Infraspec, St. Petersburg, Russia), which is also fully described in works [27,42]. This spectrometer featured a total spectral range spanning from 370 to 7800 cm^−1^ (equivalent to 1.3–27 μm), with a spectral resolution of 1.0 cm^−1^ and an absolute error of ±0.05 cm^−1^.

This investigation was conducted by measuring the infrared (IR) spectra of Nafion membranes in various salt solutions, such as LiCl, KCl, and NaCl, at different concentrations (0.1 M, 0.01 M, 0.001 M). All water-based solutions were made using deionized water with a resistivity of 18 MΩ·cm, obtained from a four-cartridge Nanopure Infinity System (Barnstead, Dubuque, IA, USA). Special attention was paid to the dynamics of the extremum in the range of valence vibrations around 5200 ± 500 cm^−1^ during the soaking and subsequent drying of the Nafion membrane at various time intervals. A time interval of 3–10 min separated each set of measurements. All measurements were conducted at room temperature (23 °C).

Preprocessing of the IR spectra involved baseline correction by identifying local minima within the specified spectral range, which were presumed to represent the underlying baseline. Furthermore, aligning these spectral minima facilitated correction for shifts induced by factors like instrument drift or fluctuations in sample preparation.

**Figure 3:**
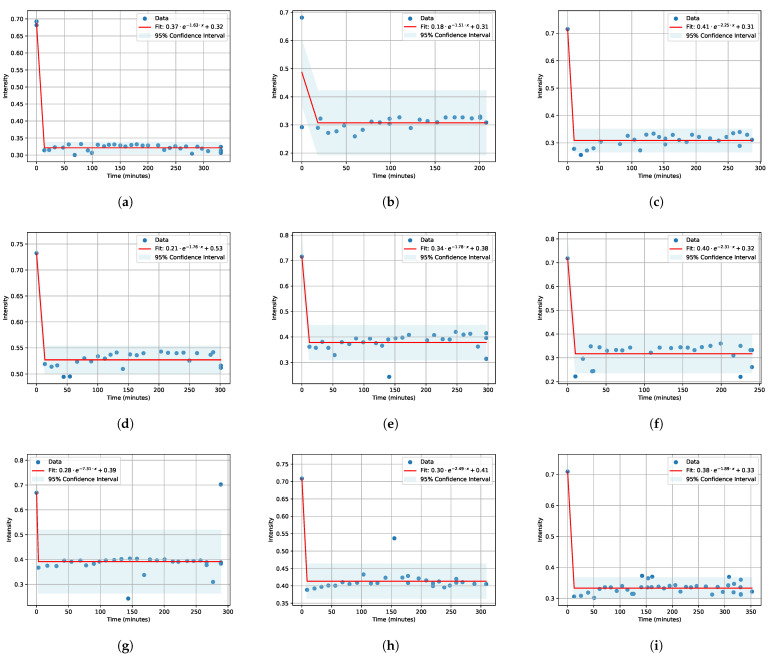
Nafion soaking kinetics for salt solutions of LiCl (**a**–**c**), KCl (**d**–**f**), NaCl (**g**–**i**) at salt concentrations of 0.1 M, 0.01 M, and 0.001 M, respectively.

**Figure 4:**
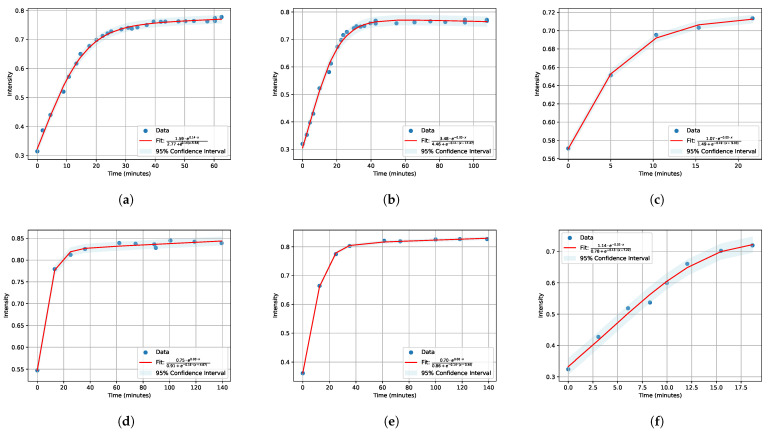
Nafion drying kinetics with saturation approximations for salt solutions of LiCl (**a**–**c**), KCl (**d**–**f**) at salt concentrations of 0.1 M, 0.01 M, and 0.001 M, respectively.

Subsequently, the obtained minimum values at different time points were plotted in graphs (Figure 3 and Figure 4) and then fitted with an exponential function; for swelling, this was a decreasing exponential, and for drying, this was a saturation curve. Fitting was conducted using a custom Python 3.10 code, specifically utilizing the module Model from the lmfit library [43]. This module allowed for the implementation of mathematical models to fit the obtained minimum values at different time points. The lmfit library provided robust tools for parameter estimation and curve fitting, enabling precise characterization of the time-dependent behavior observed in the experimental data.

Then, ML models were applied to the gathered data. Specifically, the concentration information and salt solution compositions were utilized as input features, while the coefficients of the fitted exponential functions, representing self-diffusion coefficients, served as output (predictive) features.

In modern science, there is a constant need to optimize experimental processes for more efficient data acquisition. It was necessary to conduct numerous measurements to obtain diffusion coefficients (A, B for soaking and C, D, E for drying) at various concentrations of salts and salt solutions. Each measurement required significant time—up to several days—to yield reliable results. The figures presented in our paper (Figure 3 and Figure 4) illustrate only fragments of data collected during the first 6 h of the experiment.

In light of this, we propose an approach based on machine learning [44,45], specifically neural networks (NNs) [46,47], to predict diffusion coefficients based on existing data. This approach could offer an estimate of the diffusion coefficients under various experimental conditions, helping researchers determine the necessity of conducting specific measurements.

The advantages of using NNs in this context include a high flexibility and the ability to adapt to complex nonlinear relationships between input and output data [48]. NNs can extract hidden patterns from data and make accurate predictions even in the presence of noise and incomplete data. The use of NNs in optimizing experimental processes [49] can significantly reduce the time and resources required to obtain valuable data, contributing to faster progress in scientific research.

## 3. Results

### 3.1. IR Spectra and Fitting

**Figure 5:**
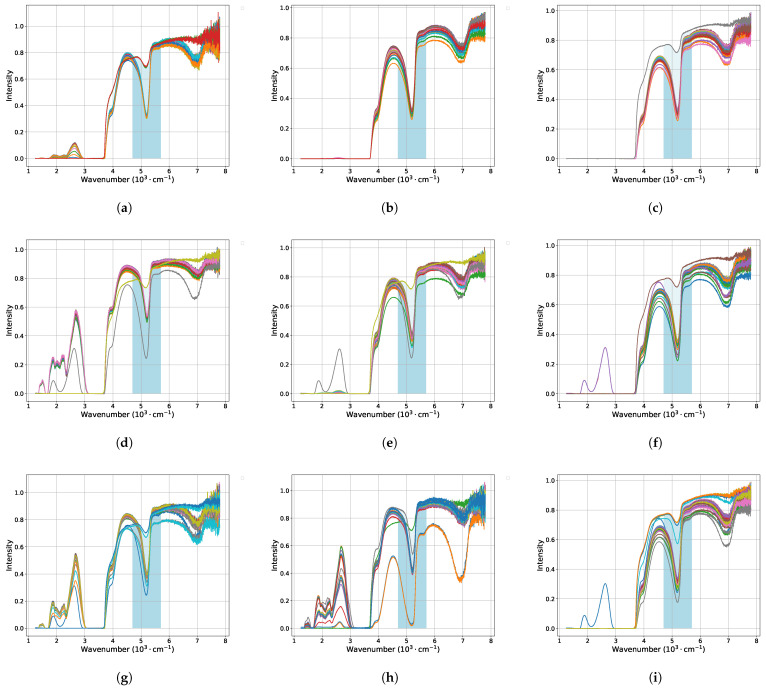
IR spectra of Nafion membranes during soaking in salt solutions of LiCl (**a**–**c**), KCl (**d**–**f**), and NaCl (**g**–**i**) at salt concentrations of 0.1 M, 0.01 M, and 0.001 M, respectively.

**Figure 6:**
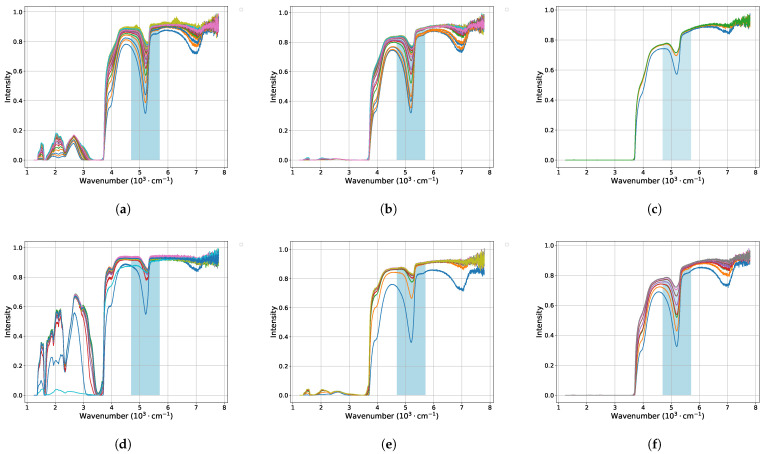
IR spectra of Nafion membranes during drying in salt solutions of LiCl (**a**–**c**) and KCl (**d**–**f**) at salt concentrations of 0.1, 0.01, 0.001, respectively.

The obtained results of IR spectra during the swelling and drying processes of Nafion membranes at various time intervals are presented in Figure 5 and Figure 6. Each graph corresponds to Nafion’s IR spectra at a specific concentration of a single soaking or drying salt.

The highlighted blue bands in these figures indicate the specific range of interest, within which the identified minima were subsequently fitted over time using exponential functions. The results of these fittings can be observed in Figure 3 for soaking, with approximation function:

(1) $$A\cdot e^{-B\cdot x}+C$$

and for drying, with approximation function:

(2) $$\frac{\tilde{A}\cdot e^{-\tilde{B}\cdot x}}{\tilde{C}+e^{-\tilde{D}\cdot \left(x-\tilde{E}\right)}}$$

in Figure 4. These functions exhibit the most robust behavior suitable for an automated approximation process.

The fitting was performed with a 95% confidence interval [50] which indicates that the range of values obtained from the exponential fitting has a 95% probability of containing the true population parameter.

The coefficients obtained from the fitting graphs are essential for determining the self-diffusion coefficients of ions diffusing into or out of a membrane sample in contact with an equilibrating solution. This approach relies on several assumptions [51]: firstly, the membrane governs the diffusion process; secondly, the self-diffusion coefficient within the membrane remains constant; and thirdly, the diffusion process is one-dimensional.

The validity of the first assumption hinges on ensuring that the concentrations of ions [52] at both boundaries of the membrane match those in the bulk solution, achieved through vigorous stirring of the membrane sample in the equilibrating solution [53]. The concentration profile of radiotracer ions diffusing into or out of the membrane is a function of both time and space, following Fick’s second law [54]:

$$\frac{\partial c}{\partial t}=D\frac{\partial^{2}c}{\partial x^{2}}$$

Here, *c* represents the concentration of ions within the membrane, *D* denotes the self-diffusion coefficient of the ion within the membrane, and *x* is the spatial coordinate.

Analytical solution of Fick’s second law is given by the expression [55,56]:

$$n\left(t_{k}\right)=n^{*}\left[1-\left(\frac{8}{\pi^{2}}\right)\left\{e^{-D_{H_{2}O}^{m}\pi^{2}t_{k}/L^{2}}+\frac{1}{9}e^{-9D_{H_{2}O}^{m}\pi^{2}t_{k}/L^{2}}+...\right\}\right]$$

where $n(t_{k})$ and $n^{*}$ represent the quantities of ions in the equilibrating solution at fixed times $t_{k}$ and $t_{\infty }$, respectively. $D_{H_{2}O}^{m}$ denotes the self-diffusion coefficient of water within the membrane, and *L* represents the thickness of the membrane.

All obtained results of approximations were compiled into Table 1 and Table 2 for clarity.

### 3.2. Neural Network (NN)-Based Model for Predicting Diffusion Coefficients

In this study, we utilized an NN to forecast diffusion coefficients using a dataset encompassing salt concentration, time (during which the approximation was conducted), initial intensity, and intensity measured in the subsequent measurements, which is also referred to as coefficient *C*. This coefficient essentially represents the average value derived from all measurements taken from the second one onwards.

We have gathered all the necessary experimental parameters, such as concentration and salt type, by conducting measurements including initial measurements, measurements of coefficient *C* (which represent the average between the second and third measurements), and measurements using time as the interval between the first and third measurement. Our goal is to predict, using a neural network, coefficients *A* and *B* of exponential decay. These coefficients reflect the long-term process of intensity change in IR measurements during the soaking of Nafion membranes in water over time. Before initiating model training, we conducted several steps of data preprocessing. Initially, we employed One-Hot encoding [57] on the ’Salt’ column to transform categorical data into binary flags, categorizing salts such as LiCl, KCl, or NaCl accordingly. Then, we scaled numerical features using standardization [58] to ensure similar value ranges.

We employed a genetic algorithm (GA) approach to optimize the parameters of the NN model [59,60] of TensorFlow [61] for predictive analysis. The aim was to develop an accurate model for forecasting the properties of interest based on the input features derived from experimental data. The methodology consisted of several key steps as follows:

1. Definition of a multi-layer NN architecture using the TensorFlow framework. The architecture comprised multiple dense layers with varying numbers of neurons and activation functions, which were treated as parameters to be optimized by the genetic algorithm.
2. GA implementation using the DEAP (Distributed Evolutionary Algorithms in Python) library. We defined the individuals as a combination of integers representing the number of neurons in each dense layer and a categorical variable representing the activation function. The GA aimed to optimize these parameters to minimize the mean squared error (MSE) loss function of the NN model.
3. GA iteratively evolved a population of candidate solutions (NN architectures) over multiple generations. Each candidate solution was evaluated by training the corresponding NN model on the training dataset and computing its MSE loss on the validation dataset. The fitness of each solution was determined by its validation MSE.
4. After a predefined number of generations, the GA selected the best-performing individual (NN architecture) based on its validation MSE. We split the data into training and testing sets in an 80/20 ratio. This architecture was then used to train a final NN model on the entire training dataset.
5. The performance of the final NN model was evaluated on an independent testing dataset to assess its generalization ability and predictive accuracy. The MSE loss on the test dataset was calculated as a measure of model performance.

**Figure 7:**
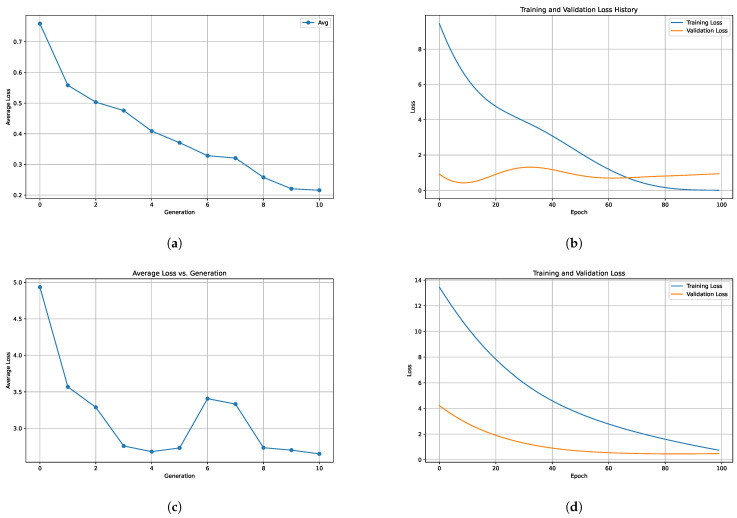
Average loss across generations during genetic algorithm optimization for predicting (**a**) A and B soaking coefficients, (**c**) $\tilde{A}$, $\tilde{B}$, $\tilde{C}$, $\tilde{D}$ and $\tilde{E}$ drying coefficients. Training and validation loss history for the best model for predicting (**b**) A and B soaking coefficients, (**d**) $\tilde{A}$, $\tilde{B}$, $\tilde{C}$, $\tilde{D}$ and $\tilde{E}$ drying coefficients.

The results of hyperparameter optimization for the neural network using a genetic algorithm (GA) are depicted in Figure 7, showcasing the performance metrics, including loss and validation loss, for the best-performing architecture obtained through optimization.

In addition to predicting the coefficients A and B which characterize the soaking process in our study, we extended our model to predict the coefficients $\tilde{A}$, $\tilde{B}$, $\tilde{C}$, $\tilde{D}$, and $\tilde{E}$ obtained from infrared (IR) spectroscopy measurements during the drying phase of Nafion membranes after soaking. This expansion allowed us to capture the dynamic changes in material properties during both the soaking and drying stages. Notably, we also employed hyperparameter optimization techniques using the GA to fine-tune the performance of our predictive model. By optimizing these parameters, we aimed to enhance the model’s accuracy and robustness, thereby improving its ability to generalize and provide reliable predictions across diverse experimental conditions.

As a result, the best model for predicting soaking coefficients included four hidden layers (64, 64, 32, 16 neurons with ReLU [62], ELU [63], sigmoid [64] and ELU activation functions [65], respectively), as well as an output layer with two neurons for predicting coefficients A and B.

The best model for predicting drying coefficients included two hidden layers (16 and 12 neurons with sigmoid and ELU activation functions, respectively), as well as an output layer with five neurons for predicting coefficients $\tilde{A}$, $\tilde{B}$, $\tilde{C}$, $\tilde{D}$, and $\tilde{E}$.

**Figure 8:**
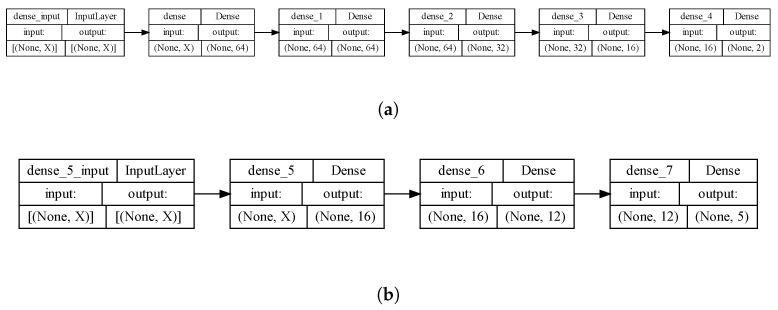
Diagram depicting the architecture of the NN for predicting diffusion coefficients (**a**) for the soaking process and (**b**) for the drying process.

The uilized architecture for both cases of NNs is shown on Figure 8. Here, the input layer’s dimension of X signifies the number of entities in the training dataset. With our dataset limited by experimental constraints, utilizing NNs for coefficient estimation becomes necessary. However, the model’s parameters relative to the dataset size raise concerns about overfitting. Balancing complexity and the dataset size is crucial for reliable predictions. Further investigation is warranted.

After compiling the model with the Adam optimizer [66] and MSE loss function [67], we trained the model on the training dataset for 100 epochs [68], using a validation set to assess performance and prevent overfitting.

At the end of training, we evaluated the model’s performance on the testing dataset and obtained the loss value. We also made predictions for values A and B based on the testing dataset, obtaining a test loss [69] of 0.44. The result is shown in Table 3.

This approach demonstrates the effective use of NNs for predicting diffusion coefficients based on input parameters. The resulting model can be used to predict the values of A and B with a high accuracy, which is an important step in diffusion research.

The performance of the optimized neural network model was compared against several other regression algorithms, including Linear Regression, Ridge Regression, Lasso Regression, Decision Tree Regressor, Random Forest Regressor, Gradient Boosting Regressor, Support Vector Regressor (SVR), and Multi-layer Perceptron Regressor (MLPRegressor). This comprehensive comparison enabled the assessment of the neural network’s predictive capabilities relative to more traditional and state-of-the-art regression techniques, providing valuable insights into its effectiveness across different modeling scenarios. The results are shown in Table 4 and Table 5.

## 4. Discussion

The derived diffusion coefficients reflect the rate at which different species, such as protons or other ions, diffuse through the membrane [78,79]. Exponential coefficients play a significant role in soaking and drying processes. In the case of soaking, particular attention is given to the coefficient *B*, while in drying, coefficients $\tilde{B}$ and $\tilde{D}$ are considered.

In fuel cells, for instance, protons must efficiently transport across the membrane from the anode to the cathode for electrochemical reactions to occur. The diffusion coefficients help quantify how effectively this transport process happens. Higher diffusion coefficients indicate faster movement of species through the membrane, which can lead to an improved cell performance, a higher power output, and a better overall efficiency.

Moreover, these coefficients can provide insights into the membrane’s structural and chemical properties. Changes in diffusion coefficients under different operating conditions or with modifications to the membrane composition can reveal how alterations affect ion transport and membrane performance. This understanding is crucial for designing Nafion membranes tailored to specific applications, optimizing their performance and addressing challenges such as durability, efficiency, and cost-effectiveness in fuel cells and other electrochemical devices.

Here are some possible research directions in the application of neural networks for predicting diffusion coefficients:

- Improving deep learning models: Research on developing more efficient and accurate deep learning models for predicting diffusion coefficients. This may involve using more complex network architectures and optimizing training parameters [80].
- Using recurrent neural networks (RNNs): Research on applying recurrent neural networks to analyze time series data, such as changes in substance concentration over time, to predict diffusion coefficients [81].
- Training on diverse datasets: Research aimed at training models on diverse and larger datasets of diffusion data [82]. This can help improve the models’ generalization ability and make them more applicable to various conditions and materials.
- Investigating the influence of material structure: Research on analyzing the influence of the material’s structure on its diffusion properties using neural networks [83]. This may involve analyzing the microstructure of the material or its chemical composition.
- Integration of Physical Models: Research aimed at integrating physical diffusion models with neural networks [84,85] to improve prediction accuracy. This can help incorporate the physical laws underlying diffusion into model development.
- Application in multiscale modeling: Research on using neural networks in multiscale diffusion modeling [86,87], allowing for the consideration of different temporal and spatial scales of the process.

These research directions can help advance our understanding of diffusion processes and develop more accurate and applicable models for their prediction.

It is imperative to acknowledge that further improvements in model accuracy necessitate a continuous influx of data [88] by performing more experiments. A larger and more diverse dataset would enable our models to capture a wider range of patterns [89] and nuances inherent in the diffusion process, thereby refining their predictive capabilities and bolstering their applicability across various experimental conditions. Also, it would mitigate the risk of overfitting and enhance the generalizability of our models, ensuring robust performance in real-world scenarios.

## 5. Conclusions

In conclusion, our study applied various machine learning approaches, including the optimization of neural network hyperparameters using a genetic algorithm, as well as employed different regressors to predict the coefficients governing the IR-based behavior of ion diffusion in Nafion membranes immersed in salt solutions during soaking and drying processes. These methodologies enabled us to enhance our understanding of the complex diffusion processes occurring within Nafion membranes and provide valuable insights into their behavior in different salt environments. Through the integration of advanced analytical techniques with computational methodologies, our research contributes to the ongoing efforts aimed at optimizing the performance of Nafion membranes in various applications, such as fuel cells and electrochemical devices. 

## Figures and Tables

**Table 1:** Obtained coefficients *A*, *B* and *C* (see Equation (1)) at various concentrations of salts in different solutions from Figure 3.

Salt	Concentration	*A*	*B*	*C*
LiCl	0.1 M 0.01 M 0.001 M	0.37 0.18 0.41	−1.63 −1.51 −2.25	0.32 0.31 0.31
KCl	0.1 M 0.01 M 0.001 M	0.21 0.34 0.40	−1.76 −1.78 −2.31	0.53 0.38 0.32
NaCl	0.1 M 0.01 M 0.001 M	0.28 −7.31 0.39	0.3 −2.49 0.41	0.38 −1.89 0.33

**Table 2:** Obtained coefficients $\tilde{A}$, $\tilde{B}$, $\tilde{C}$, $\tilde{D}$, $\tilde{E}$ (see Equation (2)) at various concentrations of salts in different solutions from Figure 4.

Salt	Concentration	$\tilde{A}$	$\tilde{B}$	$\tilde{C}$	$\tilde{D}$	$\tilde{E}$
LiCl	0.1 M 0.01 M 0.001 M	1.59 3.48 1.07	0.14 0 0	2.77 4.46 1.49	0.14 −0.11 −0.19	−5.54 −17.47 −5.03
KCl	0.1 M 0.01 M 0.001 M	0.75 0.7 1.14	0 0 −0.02	0.91 0.86 0.78	−0.16 −0.14 −0.13	−4.87 −0.44 −7.22

**Table 3:** Real vs. predicted values for test dataset of diffusion coefficients for soaking Nafion.

Real A	Real B	Predicted A	Predicted B
0.41	−2.25	0.36	−2.32
0.34	−1.78	0.35	−1.42

**Table 4:** Mean squared error (MSE) for different regressors for predicting A and B soaking coefficients.

Regressor	A	B
LinearRegression	0.825698	0.011197
Ridge	0.048564	0.127635
Lasso	0.265159	0.589168
DecisionTreeRegressor	0.012850	2.426850
RandomForestRegressor	0.138267	0.145108
GradientBoostingRegressor	0.002541	0.545394
SVR	0.012887	0.134859
MLPRegressor	0.056801	0.514195

**Table 5:** Mean squared error (MSE) for different regressors for predicting $\tilde{A}$, $\tilde{B}$, $\tilde{C}$, $\tilde{D}$, and $\tilde{E}$ drying coefficients.

Regressor	$\tilde{A}$	$\tilde{B}$	$\tilde{C}$	$\tilde{D}$	$\tilde{E}$
Linear Regression [70]	0.135808	0.000677	0.272925	0.016502	17.032002
Ridge [71]	0.146546	0.000236	0.445721	0.009340	17.608812
Lasso [72]	0.526937	0.002125	1.763845	0.003050	14.911644
Decision Tree Regressor [73]	0.070900	0.000200	0.009700	0.000650	12.932100
Random Forest Regressor [74]	0.103242	0.002025	0.992413	0.002571	15.126330
Gradient Boosting Regressor [75]	0.304507	0.000200	0.207288	0.001429	11.885889
SVR [76]	0.177954	0.006500	1.016009	0.005524	13.676221
MLPRegressor [77]	0.057762	0.055338	0.031318	0.067580	9.837034

## Data Availability

The original contributions presented in the study are included in the article, further inquiries can be directed to the corresponding author.
